# Impact of Forest Seral Stage on use of Ant Communities for Rapid Assessment of Terrestrial Ecosystem Health

**DOI:** 10.1673/031.010.7701

**Published:** 2010-06-28

**Authors:** Lynn D. Wike, F. Douglas Martin, Michael H. Paller, Eric A. Nelson

**Affiliations:** Savannah River National Laboratory, Savannah River Site, Aiken, SC, USA 29808; ^1^ Current address: Department of Biology and Geology, University of South Carolina — Aiken, University Parkway, Aiken, SC 29801; ^2^ Current address: University of Texas, Texas Natural History Collections, PRC 176 / R4000, 10100 Burnet Road, Austin, TX 78758-4445

**Keywords:** ant functional groups, pine plantation, rapid bioassessment, silviculture, southeastern USA

## Abstract

Bioassessment evaluates ecosystem health by using the responses of a community of organisms that integrate all aspects of the ecosystem. A variety of bioassessment methods have been applied to aquatic ecosystems; however, terrestrial methods are less advanced. The objective of this study was to examine baseline differences in ant communities at different seral stages from clear cut to mature pine plantation as a precursor to developing a broader terrestrial bioassessment protocol. Comparative sampling was conducted at nine sites having four seral stages: clearcut, 5 year recovery, 15 year recovery, and mature stands. Soil and vegetation data were also collected at each site. Ants were identified to genus. Analysis of the ant data indicated that ants respond strongly to habitat changes that accompany ecological succession in managed pine forests, and both individual genera and ant community structure can be used as indicators of successional change. Ants exhibited relatively high diversity in both early and mature seral stages. High ant diversity in mature seral stages was likely related to conditions on the forest floor favoring litter dwelling and cold climate specialists. While ants may be very useful in identifying environmental stress in managed pine forests, adjustments must be made for seral stage when comparing impacted and unimpacted forests.

## Introduction

The concept of “ecosystem health” is complicated and controversial (Calow 2000; Nielsen 1999; Rapport 1999; Suter 1993; Wicklum and Davies 1995), but is used here as shorthand for a complex group of related ecosystem concepts. This concept is important because it can provide information about the effects of external influences such as invasive species and disturbance and can be used to measure the rate and trajectory of change of systems that are suffering impact or being restored. Ecosystem health is also the best indicator of the success of long term environmental stewardship because it provides a baseline condition and a means for future comparison and evaluation. Ecosystem health is difficult to assess because there are many biotic and abiotic variables and no consensus as to which ones are truly indicative of ecosystem condition. It would be impossible and prohibitively expensive to measure all variables, and there are conflicting opinions as to which are most important, easily measured, and robust. One approach that avoids this controversy is bioassessment, which evaluates ecosystem health using responses of organisms within the system itself, thus integrating all aspects of the system in question.

Historically, measurements of surrogate parameters, such as water chemistry in aquatic systems, have been used in an attempt to quantify anthropogenic change. Unfortunately, pollution is frequently transient, and the effects are often missed when only water data are collected. However, communities of organisms living in such waters integrate pollutant effects over time and may show effects at low levels of chronic disturbance. Thus, for aquatic systems, especially streams, many investigators have successfully applied rapid bioassessment protocols (RBAs) that directly measure the responses of organisms affected by system perturbations (Karr 1991; Clarke 1993; Rossaro and Pietrangelo 1993; Growns et al. 1997; Thorne and Williams 1997; Guérold 2000; PalLer et al. 2005). One of the more successful is the Index of Biotic Integrity (Karr et al. 1986), which uses fish community metrics collected from a stream to rate the relative health of the stream. RBA protocols provide an integrated evaluation of ecosystem health because organisms integrate all aspects of their environment and its condition. The RBA concept has been applied with varying success to other aquatic ecosystems like slope wetlands (Paller et al. 2005), but terrestrial bioassessment methods have generally lagged behind those for aquatic systems (however, see Rosenberg et al. 1986; Kremen et al. 1993; O'Connell et al. 1998; Rodriguez et al. 1998).

In the last decade, primarily in Australia, extensive development of an RBA method using ant communities has shown promise. Ants have the same advantage for terrestrial RBAs that fish do for aquatic systems in that they are essential and ubiquitous in virtually all terrestrial ecosystems. They occupy a broad range of niches, functional groups, and trophic levels and, similar to the fishes, posses a wide range of tolerance to conditions. Within ant communities there are robust taxa that may be abundant under even the harshest impacts and sensitive taxa whose presence or absence is indicative of disturbance. As with aquatic RBAs that use feeding groups of macroinvertebrates, ants have a wide variety of functional groups (Table 1), whose abundance is useful in evaluation of ecosystem health (Andersen et al., 2004).

Ground work for useful ant RBAs has been done in Australia (Majer et al. 1984; Majer 1985; Andersen 1990; Oliver and Beattie 1996a, b; Read 1996; Andersen 1997; Majer and Nichols 1998; Lobry de Bruyn 1999;), Europe (Puszkar 1978; Gómez et al. 2003), the southwestern USA desert (Kaspari and Majer 2000), and South America (Majer 1992; Bestelmeyer and Wiens 1996; Estrada and Fernandez 1999; Osborn et al. 1999; Kalif et al. 2001). These studies have successfully evaluated restoration and recovery from a variety of anthropogenic impacts. The existing body of knowledge has transferred well to other ecoregions and could be adapted to the southeastern USA. Few studies have applied the concept to the southeastern USA (Graham et al. 2004a, b), and none have developed a regional adaptation of Andersen's (1990) original concept as done by Paller et al. (1996) with the Index of Biotic Integrity (Karr 1991). It is necessary to allocate the local ant fauna to functional groups and to evaluate metrics and characteristics to develop indices. Successful adaptation of an ant RBA would provide a cost effective, useful, and robust tool for evaluating the health of terrestrial ecosystems in the region.

Pine plantations managed for the production of forest products cover large expanses of land in the southeastern USA. Harvest cycles of 20–25 years create different seral stages that are often in close proximity. The objective of this study was to determine how sensitive ant community structure is to habitat differences among seral stages in managed pine plantations. Understanding relationships between ant community structure and habitat is an important first step in the development of ant community bioassessment protocols for the southeast.

**Table 1.**
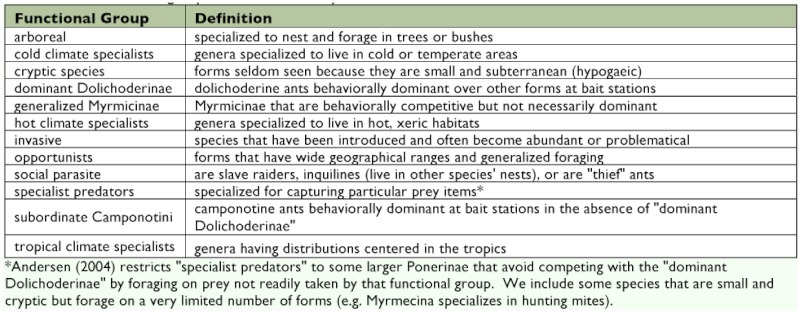
Ant functional groups as used in this study.

## Materials and Methods

### Study site

Sampling was conducted at nine sites, representing four different seral stages of pine plantation. The seral stages were clear cut, 5 year recovery, 15 year recovery, and mature planted pine stands. All sampling locations were in the Aiken County, South Carolina portion of the Sand Hill Region with seven locations on the Department of Energy's Savannah River Site (SRS) and two on private land near Windsor, SC. Four of the sites were sampled in 2006 (one clear cut, one 5 year, one 15 year, and one mature), and five sites were sampled in 2007 (two clear cut, one 5 year, one 15 year, and one mature). Each site was carefully selected to ensure that its topography and the composition and structure of its vegetation were representative of the seral stage it was intended to characterize. Soil type selection was kept as uniform as possible across the seral stages because ground dwelling ants are profoundly affected by soil structure. Rogers (1990) described the soil types at the sample locations as siliceous sand with loamy or fine-loamy components. A 200-meter transect with sample points at 10-meter intervals was established at each site, and GPS coordinates were taken at each transect end. Transects were linear where dimensions of the study plots allowed; the 2006 15-year transect had a “T” shape; the 2007 15-year, the 2007 SRS clearcut, and the 2007 5-year plot all had “L”-shaped transects, while the 2007 mature plot had a “U”-shaped sample line.

Vegetation characteristics, litter layer and surface soil characteristics were analyzed at each pitfall location. Overstory crown closure percentage was estimated, plant species present listed, and species and diameter at breast height of the individual tree nearest the pitfall were recorded. Percent cover for understory (trees or bushes greater than 2 meters in height but less than 5 meters) was also estimated, species listed, and comments about the structure noted. Percent cover of the shrub layer (woody or other perennial plants greater than 1 meter in height) was estimated, species listed, and comments noted. Percent ground cover was estimated including all seedlings, grasses, herbaceous and vine forms, lowest practical taxa listed, and comments noted. This layer was further characterized by distributing the total cover between herb, grass, and vine components. All percent cover values were ranked from 0 to 6, with 0 being no cover and 6 being 90–100% cover.

Each individual pitfall location was divided into quadrants to sample litter depth and soil organic components. A sample point approximately 1 meter from the pitfall trap was established in each quadrant, and the depth and composition of the litter layer recorded. The depth of organic matter in the soil profile was measured as an indicator of root penetration as well as organic migration from the litter. The four points at each pitfall location were averaged to describe the individual pitfall location.

### Sampling procedures

Delabie et al. (2000) and Bestelmeyer et al. (2000) extensively compared sampling methods for capturing ants for bioassessment. This lead to the development of the ALL (Ants of the Leaf Litter) protocol (Agosti and Alonso 2000) as a standard method that employs pitfall traps and litter sampling. The ALL protocol uses a 200-meter transect with sample stations at 10-meter intervals, providing 20 sample locations. Pitfall traps were placed at each sample point, and m2 litter samples were taken at odd-numbered sample points. Pitfall traps were “double cupped” and allowed to stand for 6 d to account for the “digging in” effect (Greenslade 1973). The pitfalls (9.5 cm diameter × 15 cm deep) were set by removing the inner cup and placing in the remaining cup 50 to 75 ml of a mixture of 70% ethyl alcohol and propylene glycol; traps were then allowed to collect for 3 days. In 2006, sweep net sampling was added to the ALL protocol to assess the ants on the low vegetation as well as those on the ground and in the litter. Sweep netting was conducted for one minute at each sample point; ants were removed from the nets and preserved in 70% ethyl alcohol. Litter samples were collected in 2006 and placed directly into Winkler bag funnels with chemical heat packs and hung in the field for 3 days. Ants from these samples were preserved in 70% ethyl alcohol and stored. In 2007, only pitfall traps were sampled.

### Specimen identifications

Initially ants were identified using keys from Bolton (1994), but this was inefficient because of the numerous genera in the keys that did not occur in the study area. Therefore, a list of genera was developed for the SRS using Van Pelt and Gentry (1985). In order to include all possible ant genera, Florida genera were added from Deyrup (2003) and Deyrup et al. (1989), North Carolina genera from Carter (1962), and Georgia genera from Graham et al. (2004b) and Ipser et al (2004). New identification keys were developed using this list of genera. Anatomical traits that are easy for non-specialists to identify were used whenever possible. The following sources were used to identify characters and couplets useful for a rapid identification key: Graham et al. (2004a); McGown (2007); Plowes and Patrock (2000); Van Pelt and Gentry (1985); and Mackay and Mackay (2003). This set of keys (Martin 2006) is posted at http://sti.srs.gov/fulltext/WSRC-STI-2006-00220_R1.pdf

Genera were assigned to functional groups as defined in Table 1. For the most part, functional groups were used as defined by Andersen and Majer (2004). Three other categories, arboreal species, invasive species, and social parasites, were added. As arboreal species are about twice as resistant to desiccation as ground-foraging ants (Hood and Tschinkel 1990), abundances of these forms in pitfalls may give information about local conditions. Invasive forms almost certainly either fill a vacuum where other forms are absent or have been eliminated or directly eliminate or reduce other forms through competition or predation. Social parasites probably indicate the presence of their hosts, even if these hosts were not present in our collections. In order to develop a robust tool useful to non-experts, all members of a genus were assigned to the functional group that was most inclusive of it in the collections.

### Statistical analysis

Nonmetric multidimensional scaling (NMS), a relatively assumption free ordination method, was used to identify patterns among samples based on ant genera data. All ordinations were based on Bray-Curtis similarity matrices and repeated with different random starting configurations to obtain a final solution with consistent and low stress (i.e., distortion between similarity rankings and distance rankings in the ordination plot). The number of significant dimensions (axes) in each NMS was determined with a Monte Carlo procedure that compared the stress in the ordinations with the stress in randomized data arrangements (McCune and Mefford 1999). The first ordination was based on ant presence/absence data collected using pitfall traps, litter samples (Winkler bags), and sweepnets at sites sampled during 2006, when the use of multiple sampling methods produced the most complete assessment of community composition. Spearman correlation coefficients were used to assess the influence of habitat variables on the axes produced by this ordination. The second ordination used a matrix of the number of ant genera in each function group at each sampling station, also based on the combined methods used during 2006.

The collection of a second set of seral stage samples in 2007 provided independent data that could be used to verify relationships observed in the 2006 data set. Similarities in the relationships among sample sites in the two data sets were analyzed with a Mantel test based on Bray-Curtis similarity matrices (McCune and Mefford 1999). In addition, the samples from both data sets were combined to produce a cumulative data set with two replicates for each seral stage. The significance of differences among seral stages was then tested with the Multi-Response Permutation Procedure, a multivariate, nonparametric test of differences among groups (McCune and Mefford 1999).

Because each seral stage was subsampled with a number of individual plots, it was possible to construct taxa accumulation curves and estimate the total number of ant genera in each stage with a first-order jackknife estimator (Palmer 1990, McCune and Mefford 1999). Such estimators are useful because the number of species in a sample area is generally greater than the number of observed species.

## Results

Overstory and understory canopy cover were much higher in the mature and 15 year transects (41–52% overstory coverage, 1–30% understory coverage) than in the clear cut and 5 year transects (0% overstory coverage, 0–3% understory coverage). Litter depth was also substantially greater in the 15 year and mature transects (2–4 cm) than in the earlier seral stages (0–2 cm). Shrub cover showed the opposite pattern: clear cut and 5 year transects had higher cover (16–44%) and 15 year and mature transects had lower cover (3–5%). Similarly, grass cover was higher in the clear cut and five years transects (2–15%) than in the 15 year and mature transects (<1%).

The number of samples containing each common ant genus in 2006 for all methods combined is reported in Table 2. For all methods combined (considering only genera captured 25 or more times), two genera (*Brachymyrmex* and *Dorymyrmex*) had more than 85%) of their captures in the clear cut and 5 year transects, and two genera (*Crematogaster* and *Aphaenogaster*) had more than 80% of their captures in the 15 year and mature transects. For pitfall traps (considering only genera captured 25 or more times), *Dorymyrmex* was taken 100% of the time in the clearcut and 5 year transects, and two genera (*Crematogaster* and *Aphaenogaster*) were captured more than 80% of the time in the 15 year and mature transects. For litter samples (considering only genera captured 10 or more times), no genera were primarily caught in the clear cut and 5 year transects while three genera (*Crematogaster, Myrmecina*, and *Aphaenogaster*) had 75% to 100%) of their captures in the 15 year and mature transects. For sweepnet samples (considering only genera captured 10 or more times), three genera (*Brachymyrmex, Solenopsis*, and *Dorymyrmex*) had 94–100% of their captures in the clear cut and 5 year transects, while two genera (*Crematogaster* and *Aphaenogaster*) had 85–100%) of their captures in the 15 year and mature transects.

Ants of the genus *Aphaenogaster* occurred in almost 40%) of mature pine samples, more than 50% of 15 year samples, and in three clear-cut samples (these samples were at clear cut locations that had the most residual litter. Zettler et al. (2004) state that *Aphaenogaster* ants nest in litter and organic debris so these observations are as expected.

*Dorymyrmex* were found in 95% of pitfalls in the 5 year transect and in 72% of the pitfalls in the clear cut transect (the most highly “disturbed”). In contrast, at Fort Benning, Georgia this genus was most abundant and numerically dominant in the most highly disturbed sites (Graham et al. 2004b).

**Figure 1.**
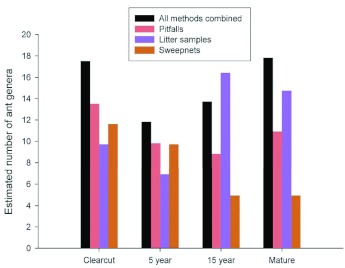
Estimated number of ant genera in four seral stages in managed pine forests in South Carolina based on 2006 data and all collecting methods. High quality figures are available online.

**Table 2.**
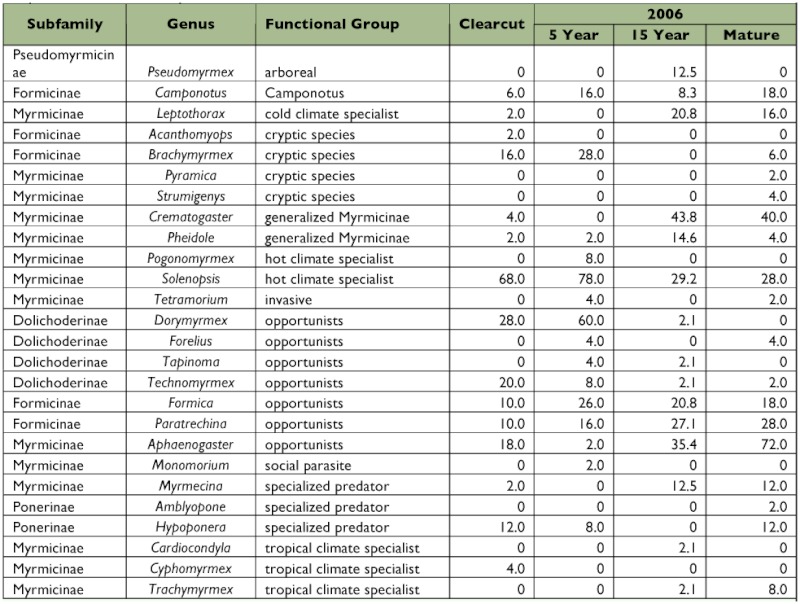
Frequency of capture of the genera of ants using pitfall, litter, and sweepnet data combined. The values reported are percent of stations occupied.

The number of ant genera estimated from the combined pitfall, litter, and sweepnet samples collected during 2006 was highest in the clearcut and mature seral stages (17.5 and 17.8, respectively), lowest in the 5 year seral stage (11.8), and intermediate in the 15 year seral stage (13.7) (Figure 1). The 2007 samples had more taxa for each treatment than did the 2006 samples. The only exception was the 2007 Windsor clear cut site where the slash was burned rather than piled up and left in the field as in the two SRS clear cut sites (2006 and 2007). This difference suggests that woody debris may constitute habitat for some ant taxa, but the sample size is too small to test this hypotheses.

Pitfall sampling alone captured most of the taxa collected by all three methods combined in the clear cut and 5 year transects but was less effective in the 15 year and mature transects (Figure 1). Litter samples exhibited the opposite pattern with greater effectiveness in the mature and 15 year transects than in the clear cut and 5 year transects. Sweepnets were effective in the clearcut and 5 year transects but collected a minority of the genera in the 15 year and mature transects. These comparisons suggest that a minimum of pitfall and litter samples were needed to adequately represent ant taxa richness across all seral stages, and sweepnet sampling was a useful adjunct sampling method in early seral stages.

**Figure 2.**
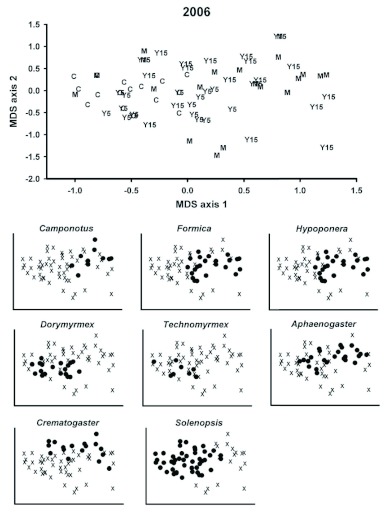
Ordination (nonmetric multidimensional scaling) of sample sites based on the presence/absence of ant genera collected in pitfall, litter, and sweepnet samples for 2006 only. Clearcut (C), 5 year (Y5), 15 year (Y15), and Mature (M) seral stages are shown. Presence is indicated by a dot and absence by an X. High quality figures are available online.

Ordination of ant presence/absence data from all sites sampled during 2006 with pitfall, litter and sweepnet samples produced two significant (p < 0.05) axes (Figure 2). The first axis separated mature and 15 year sites (with positive scores) from five year and clear cut sites (with negative scores). Litter depth, overstory canopy cover, and understory canopy cover were positively correlated (r = 0.63–0.75), and shrub cover was negatively correlated (r = -0.71) with the first axis suggesting that ant community structure responded to vegetation changes along the seral gradient represented by the study transects. Several ant genera were largely confined to late seral stage transects including *Aphaenogaster*, a litter nesting genus; *Temnothorax*, a cold climate specialist; and *Crematogaster*, a general Myrmicinae. In contrast, *Dorymyrmex* was restricted to clear cut and 5 year transects. The second axis of the ant NMS was not associated with seral stage nor was it correlated with any identified habitat variables. Two genera, *Paratrechina* and *Hypoponera*, were strongly partitioned along this axis for unknown reasons. The generalized Myrmicinae genus, *Solenopsis*, was not included in the ordination because it was present at all subsample sites.

A Mantel test indicated that the relationship between the Bray-Curtis matrices derived from the 2006 and 2007 pitfall data sets was statistically significant (r = 0.26, p = 0.001), indicating that patterns among seral stage sample sites were similar between data sets. Combining the 2006 and 2007 data sets produced a comprehensive data set (with three replicates for the clear cut sere and two replicates for each of the other seral stages) that permitted computation of the Multi-Response Permutation Procedure test. The results indicated that ant community structure differed significantly among seral stages (T = -44.9, p < 0.001), and individual pairwise comparisons showed that all seral stages were significantly different from each other (T = 7.1 to 44.9, p < 0.001 for all comparisons).

**Figure 3.**
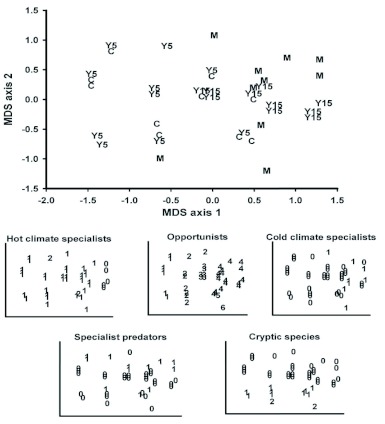
Ordination (nonmetric multidimensional scaling) of the sample sites based on the number of ant genera present in each ant functional group. Clearcut (C), 5 year (Y5), 15 year (Y15), and Mature (M) seral stages are shown. Numbers indicate the number of co-occurring genera. High quality figures are available online.

An NMS ordination based on the number of ant genera in each functional group (all sample methods combined from 2006) produced two significant (p < 0.05) axes and, like the ordination of the ant presence/absence data, separated older (mature and 15 year) and younger (clear cut and 5 year) seral stages on axis 1 (Figure 3). This separation was associated with greater numbers of opportunist and cold climate genera and an absence of hot climate specialists in some of the older seral stage sample sites. This distribution of climate specialists follows expectations since overhead canopy cover likely maintained lower ground and near-ground level temperatures in the older seral stage transects. Axis 2 was correlated with the number of cryptic and specialized predator genera for reasons that are unclear.

## Discussion

Before changes in insect communities can be generalized to changes in vascular plant communities or stress in vertebrate communities, linkage among the communities must be verified. The data indicated that vegetation related differences associated with seral stage strongly affected ant community structure. However, patterns in vascular plant and vertebrate communities often do not match patterns in invertebrate communities For example, characterization of an Austrian site based on plant communities differed from the characterization based on ant communities (Englisch et al. 2005). Differences may occur because the distribution of terrestrial invertebrates is more finely patterned than the distribution of vertebrates or vascular plants (Abensperg-Traun et al. 1996; Andersen et al. 2004).

Also, what humans perceive as environmental stress may not be stress to insects. In a European study, ant communities showed no differences among hay meadows, pastures, and silage meadows; the only factors that influenced ant communities were soil moisture and nitrogen (Dahms et al. 2005). Perhaps because of low grazing pressure (1.5 cow hectare^-1^) and infrequent mowing (1 to 3 times per year), the disturbance levels were similar among treatments. Bestelmeyer and Wiens (2001) found that livestock grazing in the semiarid American Southwest strongly affected plant communities but not ant community structure. The few noticeable changes may have been the result of soil compaction.

Poor correlations between ant communities and vascular plant or vertebrate communities seem to question the relevance of assessing ant community structure. However, ants and other soil-dwelling arthropods have very important ecological roles by contributing to soil formation and fertility (Lobry De Bruyn and Conacher 1990; Farji Brener and Silva 1995; Knoepp et al. 2000). Additionally ants are important foods for vertebrates of value. *Camponotus* and *Formica* may constitute 97% of the pileated woodpecker diet (Bull et al. 1992), while arboreal ants of the genus *Crematogaster* are the dominant food of the red cockaded woodpecker (Hess and James 1998), a species of concern in the southeastern USA.

Ant communities are certainly viable indicators of ecosystem stress where they and vascular plant communities react similarly. At Fort Benning, Georgia, disturbance by military maneuvers resulted in significant changes in the ground-foraging ant communities, but there was no measurable effect on ants living or foraging on trees (Graham et al. 2004b). Also, ants can indicate chemical pollution. Hoffmann et al. (2000) reported that ant communities were affected by dry deposition of SO2, although an examination of ant functional groups provided no additional information about stress levels.

A commonly reported pattern in ant taxon numbers related to clear cutting is fewer taxa after clear cutting followed by a period of recovery when ant taxa become more numerous than in mature stands and then a slow decline to numbers typical of mature stands. This decline is associated with canopy closure (Witford and Gentry 1981; Puntilla et al. 1991; Lough 2003). Zettler et al. (2004) reported that the loss in ant taxa following clear cutting in South Carolina lasted about 24 months. In the present study, ants exhibited relatively high diversity in both early and mature seral stages. High ant diversity in the mature seral stages was likely related to changes that favored litter dwelling and cold climate specialists. In the mature pine stands, litter was relatively thick and probably provided more insulation, thus equilibrating temperatures while reducing soil moisture loss through evaporation.

Functional groups are useful to reduce apparent complexity, identify general patterns of community structure across biogeographical boundaries, and provide ecological information about ant communities (Andersen 1997). Generally, members of a particular functional group tend to react similarly to stress (Hoffmann and Andersen 2003). Cryptic species respond to perturbances of the litter layer. In Australia, opportunists often proliferate with disturbance unless there is a dominant dolichoderine to control them, while in forested areas, fire or grazing disturbances favor increases in dominant dolichoderines and hot climate specialists (Andersen 1995). Buildup of litter in forested areas favors opportunists and reduces abundance of dominant dolichoderines and hot climate specialists. The results of this study were in partial agreement: hot climate specialists predominated in the early seral stages and cold climate specialists in the mature stands. However, opportunists were more prevalent in the older seral stages.

Other studies on ant communities in forested areas in the southeastern USA including Fort Benning, Georgia (Graham et al. 2004b) and northern Florida (Lubertazzi and Tschinkel 2003) have not recorded as much variability as was observed in this study, probably because four different seral stages were examined while these other studies included only a single more mature sere. The data from the seral stages indicated that ants responded strongly to the habitat changes that accompanied ecological succession in managed pine forests and that individual genera, as well as ant community structure, can indicate successional change. It is important to consider seral stage when developing an RBA based on ant faunas in forested locations.

## Abbreviations

**RBA**: rapid bioassessment protocol;
SRS: Savannah River Site
